# Effects of Sublethal Concentrations of Insecticides on the Functional Response of Two Mirid Generalist Predators

**DOI:** 10.1371/journal.pone.0144413

**Published:** 2015-12-07

**Authors:** Angeliki F. Martinou, Menelaos C. Stavrinides

**Affiliations:** Department of Agricultural Sciences, Biotechnology, and Food Science, Cyprus University of Technology, Arch. Kyprianos 30, Limassol, 3036, Cyprus; University of Natural Resources and Life Sciences, Vienna, AUSTRIA

## Abstract

The use of agrochemicals particularly pesticides, can hamper the effectiveness of natural enemies, causing disruption in the ecosystem service of biological control. In the current study, the effects of the insecticides thiacloprid and chlorantraniliprole on the functional response curves were assessed for two mirid predator nymphs, *Macrolophus pygmaeus* Rambur and *Nesidiocoris tenuis* Reuter. In the absence of insecticides, both predators exhibited a type II functional response when feeding on eggs of the moth *Ephestia kuehniella*. *N*. *tenuis* seems to be a more efficient predator than *M*. *pygmaeus*, as model estimated handling time was significantly lower for the former than for the latter. Residual exposure of *M*. *pygmaeus* to sublethal concentrations of either insecticide was associated with a change in the asymptote but not the type of the functional response curve. Thiacloprid seems to be the least compatible with *M*. *pygmaeus*, as it led to both a significant reduction of the attack rate and an increase in handling time. In contrast, chlorantraniliprole exposure significantly increased the handling time, but not the attack rate of the predator. Residual exposure of *N*. *tenuis* to sublethal concentrations of either insecticide did not have a significant effect on the type nor the parameters of the functional response model. The results show that pesticide residues that do not have lethal effects on beneficial arthropods can reduce prey consumption depending on predator species and on likely risks associated with toxicity.

## Introduction

Pesticides are used globally for arthropod pest suppression and play a major role in integrated pest management (IPM) strategies in many cropping systems. Insect predators are intentionally released or naturally occurring in agricultural fields and offer the important ecosystem service of biological control [1]. In crop systems where pesticides are applied, their compatibility with biocontrol agents is a major concern for IPM practitioners as it is essential for the overall agro-ecosystem resilience to be maintained.

Pesticides can cause mortality to many biocontrol agents and the assessment of acute toxicity has long been used in the evaluation of pesticide safety to natural enemies [2]. In recent years, a lot of attention has been placed on the sublethal effects of pesticides on predators, including impacts on longevity, fecundity, developmental rate, sex ratio and behavior [3–9]. However, a complete understanding of the impact of many plant protection products on the ability of predators to supress pest populations is still lacking.

Pest suppression by a predator species depends strongly on two major components of predator-prey interactions: the predator’s numerical and functional response [10,11]. The functional response is defined as the relationship between the number of prey attacked by a single predator during a given time interval and prey density. Holling [12] proposed three types of functional responses: type I, a linear rise to a plateau; type II, a curvilinear rise to a plateau; and type III, a sigmoid curve rising to a plateau which then levels off under the influence of handling time or satiation [13]. Functional response models are of interest to IPM practitioners who traditionally have tried to identify predators that impose positively density–dependent mortality on prey species (type III functional response) because such mortality is thought to stabilize prey populations [14]. Models of functional response are also employed by ethologists who wish to estimate parameters that describe predator foraging and explore their dynamics and provide a conceptual understanding of prey-predator relationships [15,16].

Among the types of functional responses, type ІІ and ІІІ have received the most attention [14], because most natural enemies show these types. Several factors can influence the functional response of predators, such as the host plant [17–19], intra or interspecific interactions [20–22], presence of alternative prey [23], predator or prey size [24,25] and pesticide exposure [26–30]. Yet, the effects of pesticides on the functional response of many important natural enemies have not been investigated.

The zoophytophagous predators *Macrolophus pygmaeus* Rambur and *Nesidiocoris tenuis* Reuter (Hemiptera: Miridae) are native in the Mediterranean region and have been commercially mass produced and successfully released in temperate and Mediterranean crops including tomato and other vegetables. Both species are used for the control of pests, such as whiteflies, thrips, aphids, mites and eggs of Lepidoptera [31–33], including the moth *Tuta absoluta* Meyrick (Lepidoptera: Gelechiidae), a pest that invaded Europe in 2006 and continues to spread in Afro-Eurasia [34,35]. However, the functional response of the predators to eggs of Lepidoptera, a major pest group, has never been compared. Furthermore, no information exists on the impact of pesticides on their functional response parameters.

In the present study, we developed functional response curves for *M*. *pygmaeus* and *N*. *tenuis* nymphs feeding on eggs of the moth *Ephestia kuehniella* Zeller (Lepidoptera: Pyralidae), a factitious prey. In addition, we investigated the effects of thiacloprid and chlorantraniliprole, two insecticides with different mode of action that are commonly used in vegetable crops, on the functional response parameters of the two predators.

## Materials and Methods

### 2.1 Pesticides

We tested the insecticides thiacloprid (CALYPSO 480 SC®- Bayer CropScience, Leverkusen, Germany) and chlorantraniliprole (CORAGEN®- DuPont Crop Protection, Wilmington, DE, USA), two products that are registered for use in tomato crops and other vegetables against several pests. Chorantraniliprole is used against Lepidoptera, while thiacloprid is used against Lepidoptera and Hemiptera. Thiacloprid is a neonicotinoid insecticide that acts as an agonist on the insect nicotinic acetylcholine receptor [36]. Chlorantraniliprole is a newer product, an anthranilic diamide that activates the ryanodine receptor, releasing stored calcium from muscle cells which leads to impaired regulation of muscle contractions [37]. Each pesticide was sprayed at half of the highest recommended label rate, at 20.00 and 72.00 mg a.i. / lt for chlorantraniliprole and thiacloprid, respectively. Testing of concentrations below the recommended field rate simulates exposure of the predators to pesticide residues in the field at several days / weeks following spray application because of the degradation of the active ingredient [38,39]. Preliminary experiments established that the rates used in the current study were sublethal, as they did not cause short-term mortality to either predator.

### 2.2 Insect Rearing

*N*. *tenuis* and *M*. *pygmaeus* and *E*. *kuehniella* eggs used as prey were provided by Koppert, Netherlands. *E*. *kuehniella* eggs are commonly used in biological control research to study predation capacity and other aspects of predator behavior (e.g. [9]). The predators were cultured in the laboratory in controlled conditions at 25±1°C, 65% RH and 16:8 L:D photoperiod. *E*. *kuehniella* eggs were kept at 10°C until use in experiments. Each species was kept separately in a tent-like polyester cage 61 x 61 x 61cm (61 cm- Bugdorm type Bioquip®, Rancho Dominguez, CA, USA) with twelve 6–8 week-old potted tomato plants variety Hybrid Brillante F1 (Hazera Genetics Ltd., 79837, Israel) and *E*. *kuehniella* egg prey. Fifth instar predator nymphs (F1) were collected from the cages and they were placed individually in Petri dishes with a piece of wet cotton wool and allowed to starve for 12 h prior to the experiment. Fifth-instar nymphs were used for the tests as they are more exposed to pesticide residues than adults that can fly off sprayed plants.

### 2.3 Experimental set up

Petri dishes (9 cm in diameter) were used as experimental arenas for the functional response studies. We opted for the use of Petri dishes rather than living plants as a testing substrate because both predators are zoophytophagous and differential plant feeding in response to pesticide exposure [9] could affect prey consumption and act as a confounding factor in functional response modelling. Three openings, 0.5 mm in diameter, were made at the lid covers for ventilation. A Potter spray tower (Burkard Manufacturing Co., Rickmansworth, UK) was used for the spray application on the Petri dish and the lid cover with the air pressure set at 1000 kPa. The spray volume per application was 1 ml of pesticide solution which resulted in a spray deposit of 2.55 mg/cm^2^ similar to what is recommended for bioassays according to the IOBC Working Group “Pesticides and Beneficial Organisms” [2]. Control Petri dishes were sprayed with distilled water. Predators and egg prey were not sprayed. After spraying, the Petri dishes and their lids were allowed to dry out for 24 h in the laboratory at 25±1°C, 65% RH.

*E*. *kuehniella* eggs were placed in the Petri dishes 24 h after spraying at the following densities: 4, 8, 16, 32, 64, 128, with the aid of a wet (size 0) paint brush. A piece of wet cotton wool was also placed in the Petri dish. An individual fifth instar nymph of either *N*. *tenuis* or *M*. *pygmaeus* was transferred in each Petri dish, and was allowed to forage for 24 h, after which it was removed and the consumed eggs were counted. Both predators feed by piercing and sucking, leaving the consumed eggs looking desiccated and shrivelled. Each density was replicated 10 times for each species and for each of the two pesticide treatments and the control. All experiments were carried out at 25±1°C, 65% RH and a 16:8 L:D photoperiod.

### 2.4 Data analysis

Each nymph represented a single replicate, a common approach in functional response studies that is employed to avoid underestimation of uncertainty for model parameters (e.g. [22,27,40,41]). The type of the functional response was determined by fitting a logistic regression of proportion of prey consumed versus prey offered according to Trexler et al. [40]. Briefly, the type of the curve is determined based on the value of the coefficients of the following quadratic polynomial function fitted to the data [41]:

$$\frac{Ne}{No}=\frac{\text{exp}(P_{o}+P_{1}N_{0}+P_{2}N_{0}^{2}+P_{3}N_{0}{}^{3})}{\left[1+\text{exp}(P_{0}+P_{1}N_{0}+P_{2}N_{0}{}^{2}+P_{3}N_{0}{}^{3})\right]}$$

*Ne* is the number of prey consumed, *No* the initial prey density and *P*_*0*_, *P*_*1*_, *P*_*2*_, and *P*_*3*_ the intercept, linear, quadratic and cubic coefficients, respectively, estimated using the method of maximum likelihood. If *P*_1_>0 and *P*_2_<0, the proportion of prey consumed is positively density dependent, thus describing a type III functional response. If *P*_1_<0, the proportion of prey consumed declines monotonically with the initial number of prey offered, thus describing a type II functional response [25,41].

Type II models found to explain the data best (see Results) were fitted to the data using non-linear least squares. The type II functional response model is described by the equation: *N*a = *aNT*_t_ / (1 + *aNT*_h_), where *N*a is the number of prey attacked, *a* is the rate of successful attacks, *N* is the prey density, *T*_t_ is the total available time and *T*_h_ is the handling time. Individual data points were used in the analyses in order to avoid underestimates of SE of parameter estimates [42]. Significant differences between parameters of the functional response models for different treatments were tested with the superposition of 95% confidence intervals. Mean values of *T*_h_ were used to calculate maximum attack rate defined as *T/T*_h_ [43], which represents the theoretical maximum number of prey that can be attacked by a predator during the time interval considered. All analyses were carried out using the non-linear least squares (nls) package in R version 2.14.2 [44].

## Results

**Fig 1:**
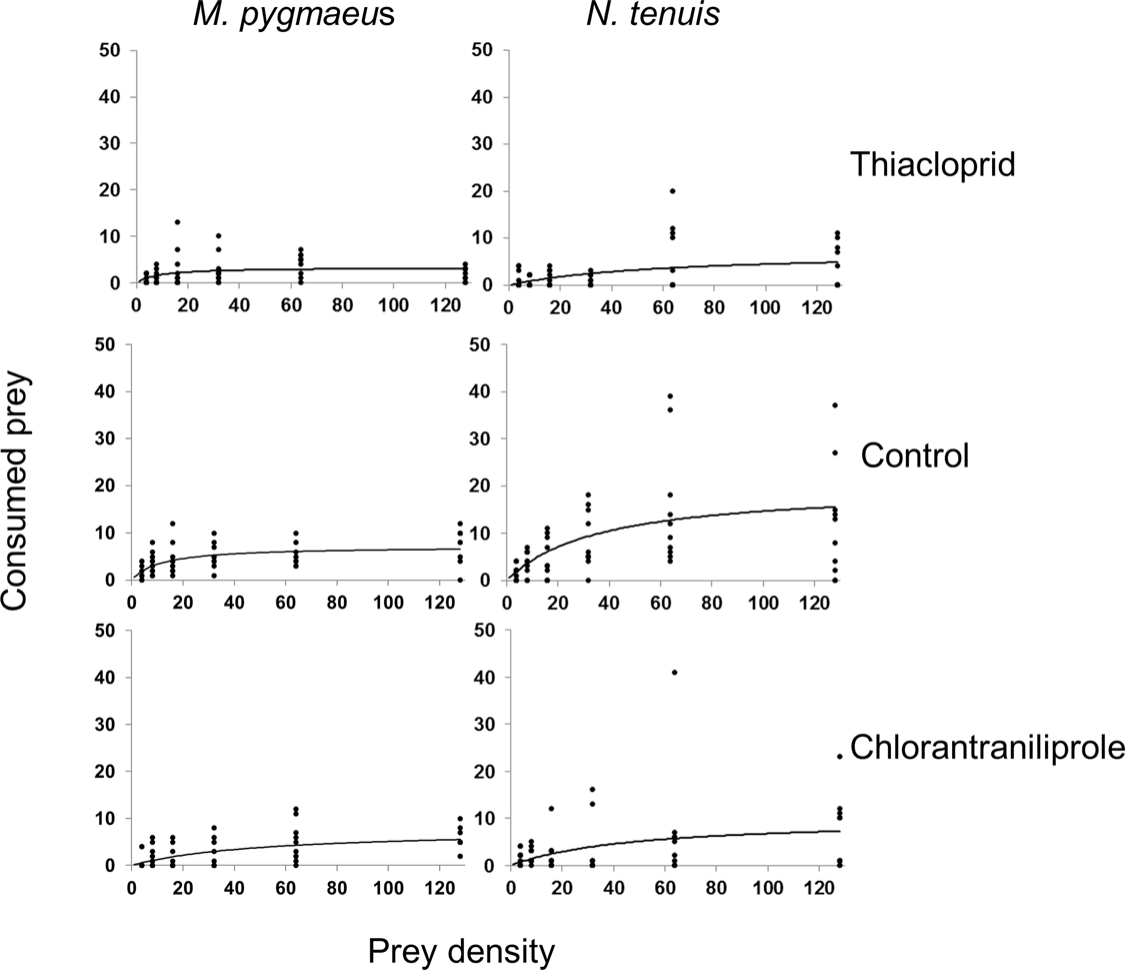
Type II functional response models for *M*. *pygmaeus* and *N*. *tenuis* for the two pesticide treatments and the control.

For both predators, and both pesticides and control the linear term *P*_*1*_ was lower than 0 (*P*<0.001, data not shown), indicating a type II functional response (Fig 1). The model parameters for the type II functional response are shown in Table 1. In control treatments, the attack rate was similar between the two predators, but model estimated handling time was significantly higher for *M*. *pygmaeus* than for *N*. *tenuis* (Table 1). As a result, the maximum attack rate was almost three times as high for *N*. *tenuis* than for *M*. *pygmaeus* (Table 1). The variation in the number of eggs consumed at each density was higher for *N*. *tenuis* than for *M*. *pygmaeus* (Fig 1, S1 Dataset).

**Table 1:** Parameters for type II functional response models (mean, 95% CI)* for *M*. *pygmaeus* and *N*. *tenuis*. RSS is the residual sum of squares of the model.

Predator	Treatment	Attack rate (a)	Max. attack rate (T/Th)	Hand. time (T_h_)	RSS
***M*. *pygmaeus***	Control	0.63 (0.56–0.70) Aa	7.3	0.14 (0.10–0.17) Ab	697.4
	Thiacloprid	0.39 (0.34–0.43) b	3.5	0.30 (0.19–0.45) a	437.5
	Chlorantraniliprole	0.15 (0.06–0.37) b	7.8	0.13 (0.04–0.21) b	422.9
***N*. *tenuis***	Control	0.56 (0.25–1.44) Aa	20.0	0.05 (0.03–0.09) Ba	3528.0
	Thiacloprid	0.12 (0.04–0.53) a	7.0	0.14 (0.00–0.33) a	795.6
	Chlorantraniliprole	0.21 (0.06–1.79) a	10.0	0.10 (0.00–0.24) a	2420.0

*Different capital letters denote significant differences in model parameters between species in control and low case letters denote significant differences among model parameters for the different treatments within species.

Although pesticide exposure did not alter the type of the functional response, it altered the asymptote of the curve, with a tendency towards lower asymptotes in pesticide treatments compared to the control (Fig 1). Model estimated handling time for *M*. *pygmaeus* was significantly higher for thiacloprid than for the control or chlorantraniliprole (Table 1). There were no significant differences in handling time between chlorantraniliprole and control. The attack rate for *M*. *pygmaeus* was significantly higher for control than for thiacloprid or chlorantraniliprole, but no statistical difference was detected between the two insecticides. Maximum attack rate values for *M*. *pygmaeus* were similar for control and chlorantraniliprole, and substantially lower for thiacloprid (Table 1).

Although model estimated handling time for *N*. *tenuis* was lower in control than for thiacloprid or chlorantraniliprole, differences were not significant (Table 1). The attack rate was higher in control than for either pesticide, but not significantly so. Maximum attack rate for *N*. *tenuis* was higher for the control than for thiacloprid or chlorantraniliprole.

No predator mortality in any treatment was observed during the 24 h observation period.

## Discussion

Both predators exhibited a type II functional response when feeding on eggs of *E*. *kuehniella*. The type II functional response model employs a decelerating predation curve that reaches a plateau as prey density increases, a destabilizing factor in prey-predator dynamics [14]. In previous research, *M*. *pygmaeus* exhibited a type II functional response curve when feeding on aphids [45] and both predators exhibited a type II response when whitefly instars or *T*. *absoluta* eggs were offered as prey [46,47].

*N*. *tenuis* seems to be a more efficient predator of *E*. *kuehniella* eggs than *M*. *pygmaeus* (Fig 1, Table 1), as handling time for the former was lower than that for the latter in control treatments. Mollá et al. [48] showed recently that *M*. *pygmaeus* fifth instar nymphs can consume approximately 10 eggs of *E*. *kuehniella* per day, whereas *N*. *tenuis* consume close to 15 eggs per day, figures similar to the ones reported in the current study. Data in Urbaneja et al. [31] also suggest that *N*. *tenuis* is somewhat a more efficient predator than *M*. *pygmaeus* when feeding on eggs of *T*. *absoluta*. Differences in predation rate, however, may be influenced by prey species and Lambropoulos et al. [46] found no difference in the consumption rates of the two predators when whitefly instar nymphs were used as prey.

Per capita predation efficiency was reduced on pesticide treated patches for *M*. *pygmaeus*, but not significantly so for *N*. *tenuis* (Fig 1, Table 1). Exposure of *M*. *pygmaeus* to either pesticide was associated with a change in the asymptote but not the shape of the functional response curve. The lower asymptote of the functional response curve where pesticides are applied is an indication of decreased predation efficiency, either because the predator attacks less prey or because of a decreasing searching time. The total time of the functional response has two components (searching time *Ts* + handling time *Th*) and an increase in the handling time results in a decrease in the time available for prey searching. Similar changes in the functional response of predators have been observed in cases of abiotic stressors, such as pesticides [29,30,49–51], biotic interactions such as intraguild predation [22] and mutual interference [21].

Thiacloprid seems to be the least compatible with *M*. *pygmaeus* as it led to both a significant reduction of attack rate and an increase in handling time (Table 1 and Fig 1). In contrast, chlorantraniliprole exposure affected the handling time but not the attack rate of the predator. In a previous study, *M*. *pygmaeus* exposed to the maximum field rate of thiacloprid residually and orally exhibited an increase in resting time and preening behavior and were not able to consume prey eggs [9]. Chlorantraniliprole exposure in the same study decreased the time spent feeding from the plant, but not predation efficiency. Thiacloprid, a neonicotinoid insecticide that acts on the acetylcholine receptors of the nervous system of Hemipterans and other pests [36], seems to be a more potent disruptor of behavioral responses of *M*. *pygmaeus* than chlorantraniliprole, an insecticide acting on the ryanodine receptors of muscle cells in insects [37]. The testing of insecticide concentrations that were below the field recommended rate suggests that the ability of *M*. *pygmaeus* to control pests may be affected even if it is released several days after spray application, when pesticide residues have started to dissipate [52]. However, dissipation rates of pesticides depend on a variety of factors, including plant species and environmental conditions [53], and therefore further experiments are needed to estimate the dissipation rates for the two pesticides for tomato plants.

*N*. *tenuis* could be more resilient to pesticide exposure than *M*. *pygmaeus*, as neither thiacloprid nor chlorantraniliprole had a significant effect on its functional response parameters (Table 1). While more studies are needed to investigate the toxicity and behavioral effects of pesticides to *N*. *tenuis*, higher resilience to commonly used products than *M*. *pygmaeus* may explain its prevalence in tomato fields in Cyprus [54].

Our results showed that pesticide residues that do not have lethal effects on beneficial arthropods can reduce a predator’s consumption of prey depending on predator species and on likely risks associated with toxicity. Although the reduction in predation in pesticide treated patches could be due to the sublethal effects of pesticides, the reduction in prey consumption in an environment that yields low return may obviously be an advantage for the predator, as it minimizes exposure to the pesticide. The preference of predators between pesticide treated and non-treated prey patches could be further tested in choice experiments. Future studies could also assess the impact of pesticides on functional response of the two predators on treated plants, however, interpretation of the results may be confounded as both species can feed on plant sap. While the current laboratory study offers important insights on sublethal effects of pesticides on biological control, additional field-based studies are needed to fully understand pesticide impacts on predation capacity of natural enemies.

Through modeling of the functional response of predators in the current study, we show that *N*. *tenuis* seems to be a more effective predator of lepidopterous eggs than *M*. *pygmaeus*. In addition, we found that two commonly applied pesticides for the control of insect pests can interfere with *M*. *pygmaeus* foraging over a wide range of prey availability at relatively low pesticide application rates. Functional response models can be used as a tool to assess the effects of chemicals on foraging efficiency of beneficial species in order to recommend or not the use of a certain product in IPM programs.

## Supporting Information

S1 Dataset Raw data for *Macrolophus pygmaeus* and *Nesidiocoris tenuis* functional response study. (XLSX)
